# Confirming Parkinson Disease Diagnosis: Patterns of Diagnostic Changes by Movement Disorder Specialists

**DOI:** 10.1155/2022/5535826

**Published:** 2022-05-09

**Authors:** Marzieh Keshtkarjahromi, Danielle S. Abraham, Ann L. Gruber-Baldini, Katrina Schrader, Stephen G. Reich, Joseph M. Savitt, Rainer Von Coelln, Lisa M. Shulman

**Affiliations:** ^1^MedStar Health, Internal Medicine Residency Program, Department of Medicine, Baltimore, MD, USA; ^2^University of Pennsylvania, School of Medicine, Department of Neurology, Philadelphia, PA, USA; ^3^University of Maryland, School of Medicine, Department of Epidemiology and Public Health, Baltimore, MD, USA; ^4^University of Maryland, School of Medicine, Department of Neurology, Baltimore, MD, USA

## Abstract

**Background:**

The American Academy of Neurology Parkinson Disease (PD) quality measures include an annual diagnostic review.

**Objective:**

To investigate the frequency and pattern of changes in diagnoses between PD and other causes of parkinsonism.

**Methods:**

This prospective longitudinal cohort study included consented patients diagnosed with PD at least once and a minimum of two times at the Movement Disorders Center between 2002 and 2017. Movement disorder specialists confirmed and documented diagnoses at every visit. Longitudinal changes in diagnoses were identified across visits.

**Results:**

Of 1567 patients with parkinsonism, 174 had non-PD parkinsonism with no change over time. Of 1393 patients diagnosed with PD at least once, 94% (*N* = 1308) had no change of diagnosis over time and 6% (*N* = 85) had a change of diagnosis including PD ⟷ drug-induced parkinsonism (DIP) (27.1%), PD ⟷ multiple system atrophy (MSA) (20.0%), PD ⟷ progressive supranuclear palsy (PSP) (18.8%), PD ⟷ Lewy body dementia (DLB) (16.5%), PD⟷ vascular parkinsonism (9.4%), more than two diagnoses (4.7%), and PD ⟷ corticobasal syndrome (CBS) (3.5%). The direction of diagnostic switches was as follows: PD ⟶ other parkinsonism diseases (36.5%), other parkinsonism diseases ⟶ PD (31.8%), and 31.8% of multiple switches. There were no significant differences in duration of follow-up, age at first visit, gender, race, marital status, education, income, cognition, or employment between the stable and unstable groups. Diagnostic change was associated with greater PD severity and greater medical comorbidity.

**Conclusion:**

Over a 15-year period, movement disorder specialists changed their clinical diagnosis of PD in 6% of patients. The most common diagnostic switches, to or from PD, were DIP, MSA, PSP, and DLB. This study describes routine clinical diagnostic patterns in the absence of pathologic confirmation. The presence of diverse diagnostic changes over time underscores the value of confirming PD diagnosis.

## 1. Introduction

The American Academy of Neurology PD quality measures include the need for annual diagnostic review [1]. Reasons for annual diagnostic review include the challenges of diagnosis, especially early in the disease course, and previous reports showing that changes in diagnosis are seen in 33% of patients after a median follow-up of 29 months [2]. In addition to PD, other causes of parkinsonism include drug-induced parkinsonism (DIP), vascular parkinsonism, and neurodegenerative conditions including progressive supranuclear palsy (PSP), multiple system atrophy (MSA), corticobasal syndrome (CBS), and dementia with Lewy bodies (DLB). Differentiating PD from other causes of parkinsonism, based on clinical diagnostic criteria, is important for management and prognosis [3, 4]. Accurate diagnosis requires a thorough history, comprehensive neurological examination, targeted diagnostic investigations, assessment of medication response, and reassessment of diagnosis over time.

Previous studies investigated the accuracy of clinical diagnosis of PD with pathological confirmation. Rajput and colleagues reported a clinical-pathologic correlation of 65% in patients at initial visit, rising to 76% after years of follow-up [5]. A systematic review and meta-analysis of clinical-pathologic correlation studies showed the diagnostic accuracy of movement disorder specialists to be 80% at initial assessment, rising to 84% with follow-up [6]. This prospective longitudinal study investigates clinical diagnoses of parkinsonism (without pathologic confirmation) made by movement disorder specialists at the University of Maryland Movement Disorders Center over a 15-year period. This study relied on data from the Center's Health Outcomes Measurement (HOME) study that requires neurologic confirmation with documentation of patient diagnosis at every office visit. The aim of this study is to investigate the frequency of diagnostic changes between Parkinson disease and other types of parkinsonism in routine clinical practice.

## 2. Methods

Study subjects were diagnosed with PD by a movement disorder specialist at the University of Maryland Parkinson Disease and Movement Disorders Center between 2002 and 2017. All movement disorder patients were asked to participate in the HOME study, and 70% of the PD patients completed informed consent as approved by the University of Maryland Institutional Review Board. To investigate diagnostic switches, eligible patients needed two or more diagnostic assessments. The study population comprises patients diagnosed with idiopathic PD by a movement disorder specialist at the University of Maryland Parkinson Disease and Movement Disorders Center (UMPDMC), applying the UK Parkinson Disease Society Brain Bank Clinical Diagnostic Criteria [7]. The diagnosis of PD or other forms of parkinsonism was made based on established clinical criteria. The data were collected prospectively, and longitudinal changes in diagnoses between PD and other forms of parkinsonism were identified across all office visits. The diagnosis of PD or other forms of parkinsonism was made based on established clinical criteria. At follow-up visits, movement disorder specialists routinely confirm and document the diagnosis on a specific physician data-entry form, based on changes in clinical symptoms, response to dopaminergic medication, and results of diagnostic tests. A diagnosis is entered on the form when the movement disorder neurologist has confidence in the diagnosis based on clinical judgment. When the treating neurologist does not have confidence to make a diagnosis, no diagnosis is entered. The collected data from eligible patients included the following: demographics, duration of parkinsonism at initial visit, years of follow-up, initial Unified PD Rating Scale (UPDRS) scores, use of levodopa or other dopaminergic medication at the initial and last visit, change of the treating neurologist, diagnosis at each visit, and medical comorbidities. Comorbidities were measured with the Cumulative Illness Rating Scale-Geriatrics version (CIRS-G) total score [8].

### 2.1. Statistical Analysis

Simple descriptive analyses of the number of patients with diagnostic changes and by types of switches are presented as numbers and percentages. Student's *t*-tests (for continuous variables) and chi-square tests compared those with diagnostic changes (unstable group) to those without changes (stable group).

## 3. Results

During 15 years of data collection (2002–2017), 1567 unique consented patients with parkinsonism were seen at the University of Maryland Movement Disorder Center. 174 (11.1%) had non-PD parkinsonism with no change in diagnosis over time. 1393 patients were diagnosed with PD at least once either at the initial visit or during follow-up visits. The mean age at the first visit of the PD group was 70.3 (SD = 10.3) years, with 63.9% male and 36.1% female. The mean duration of parkinsonism at the first visit was 4.5 (5.2) years and at follow-up was 5.4 (3.8) years. The initial CIRS-G total score was 4.9 (3.7), initial UPDRS total score was 38.7 (18.4), and initial UPDRS motor score was 26.2 (12.6). Among all patients with parkinsonism seen by movement disorder specialists over a 15-year period (*n* = 1567), 1308 (83.5%) had a PD diagnosis with no change in diagnosis over time (stable group). Among patients diagnosed with PD at least once (*n* = 1393), a change in PD diagnosis occurred in 6.1% of patients and the most common cause of a change in diagnosis was DIP.

Demographics and disease features were compared between the stable (no diagnosis change) and unstable groups (diagnosis change) to investigate predictors of diagnostic switches (Table 1). Changes in diagnosis were associated with greater PD severity and medical comorbidity at the initial visit. The stable group had a longer duration of parkinsonism than the unstable group (4.6 (5.3) vs. 3.1(5.0) years; *p* < 0.05). The unstable group had higher medical comorbidity than the stable group (CIRS-G score 6.4 (4.0) vs. 4.8 (3.6): *p* < 0.001). The unstable group also had higher total and motor UPDRS scores at the initial visit (total: 43.6 (18.3) vs. 38.4 (18.4), *p*=0.02; motor: 29.8 (12.4) vs. 26.0 (12.6), *p* < 0.01). These UPDRS differences between the unstable and stable diagnostic groups exceeds the minimally clinically important difference (CID) previously reported by our group, where the minimal CID is 4.3 points on the total UPDRS and 2.5 points on the motor UPDRS [9]. There were no significant differences (*p* > 0.05) in the following features between the stable and unstable groups: years of follow-up, age at the first visit, gender, race, marital status, education, income, employment, use of levodopa or other dopaminergic medications at baseline, cognitive assessment at the initial visit, or change of the treating neurologist. The presence of motor fluctuations or dyskinesias at baseline reduces the likelihood of a change of diagnosis (*p* < 0.01 and *p* < 0.001). However, only 28.2% had fluctuations and 19.5% had dyskinesias at the initial office visit.

**Figure 1:**
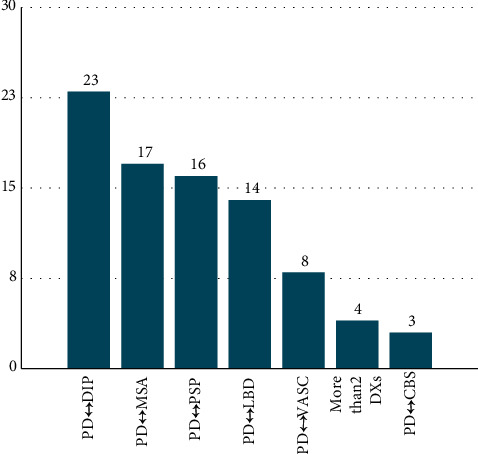
Changes in diagnosis in the unstable group (N = 85).

In the unstable group (*n* = 85), the changes in diagnosis were as follows: 23 patients (27.1%) had a switch between PD and DIP, 17 patients (20.0%) between PD and MSA, 16 patients (18.8%) between PD and PSP, 14 patients (16.6%) between PD and DLB, 8 patients (9.4%) between PD and vascular parkinsonism, 4 patients (4.7%) between more than 2 other diagnoses, and 3 patients (3.5%) between PD and CBS (Figure 1). The causes of drug-induced parkinsonism included neuroleptics (*n* = 7), metoclopromide (*n* = 5), valproic acid (*n* = 4), lithium (*n* = 3), and prochlorperazine (*n* = 1).

**Figure 2:**
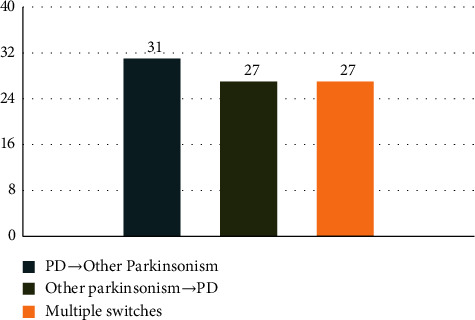
Direction of diagnostic switches (N = 85).

Patients were more likely to switch from PD to another form of parkinsonism (*n* = 31, 36.5%) than to switch from another parkinsonian diagnosis to PD (*n* = 27, 31.8%). Notably, 27 patients (31.8%) had not just one but multiple switches during follow-up visits with no clear pattern of the type or direction of the switches. In the group with multiple switches, 25 patients had 3 to 4 visits with different diagnoses and 2 patients had 2 diagnoses at the same visit (Figure 2). When we compared characteristics between these three patterns of diagnostic changes (from PD, to PD, or multiple switches), the only significant difference was employment status, with a greater number of employed patients in the group that changed from PD to parkinsonism (*p*=0.04; Table 2).

A post hoc analysis was performed to clarify the relationship between the timing of diagnostic change and disease duration. This new analysis showed that among those with diagnostic change, 43% of the changes occurred between 0 and 6 years following symptom onset. However, some diagnostic changes (16%) occurred much later, even 15 years following symptom onset. Therefore, the frequency of diagnostic change declines over time following the diagnosis. This pattern was less clear when we analyzed the entire sample (with and without diagnostic change), dividing the total sample into quartiles based on time since their baseline visit at our center. Among those seen for less than 850 days (2.3 years; *n* = 347), 6.6% had a diagnostic change. This percentage was comparable in those seen for a period of 850–1639 days (up to 4.5 years; *n* = 349, 6.3%) and those seen for 1640–2859 days (up to 7.8 years; *n* = 350, 6.6%). Among those seen for the longest period, 2860–5197 days (up to 14.2 years; *n* = 347), 4.9% had a diagnostic change. Therefore, diagnostic changes continued to occur even at 10+ years following the initial visit.

## 4. Discussion

This prospective longitudinal review of 15 years of clinical practice at a tertiary movement disorders center shows that movement disorder specialists changed their clinical diagnosis of PD in 6.1% of patients who were diagnosed with PD at least once, while 93.9% of patients diagnosed with PD had no changes in diagnosis over 5 years of follow-up. There was no difference in demographics between the stable and unstable diagnostic groups. Previous studies report that 10 years of follow-up increases PD diagnostic accuracy [6, 10–12].

Pathologic confirmation is rare in clinical practice. Therefore, clinicians must rely on the initial history and neurological exam, followed by the valuable data that are collected during follow-up visits, including symptomatic progression, response to treatment, and investigative test results. Our study aims are different than the gold standard of clinical-pathologic correlations and instead investigates the day-to-day reality of clinical practice by movement disorder specialists. Our study corroborated the importance of time since diagnosis, since a longer duration of parkinsonism was associated with a reduced risk of misdiagnosis. Other PD features associated with diagnostic changes were greater disease severity and greater medical comorbidity at the initial visit.

The explanation for these findings is likely to be that patients with fewer years of parkinsonism have less time to develop atypical signs and symptoms. However, the combination of a shorter duration of parkinsonism with greater disease severity is also consistent with the relatively rapid disease progression seen in atypical parkinsonism. Diagnostic changes tended to occur in the early years following symptom onset, although changes in diagnosis continued even at 10+ years of symptom onset. Patients in the unstable diagnostic group were found to have greater medical comorbidity than in the stable group. Comorbid medical conditions may obscure the clinical picture of PD and increase the likelihood of misdiagnosis. Comorbidities may also result in the administration of medications associated with drug-induced parkinsonism.

There was no significant difference in the use of levodopa and other dopaminergic medications at baseline between the stable and unstable groups. Of the patients with changes in diagnosis, more patients had a switch from PD to another diagnosis (36.5%) rather than the reverse (parkinsonism to PD: 31.8%). Emerging historical and clinical features, including poor levodopa response, are likely to favor the change from a diagnosis of PD to other diagnostic categories.

A prospective clinicopathologic study by Rajput et al. showed that 65% of patients diagnosed with PD at their first visit had Lewy body pathology [5]. After 12 years of follow-up, the diagnosis of PD was pathologically confirmed in 76% of patients. Another clinicopathologic study by Adler et al. reported 26% diagnostic accuracy of PD at the initial visit, reaching 53% in patients during the first 5 years of disease duration and 88% in patients with greater than 5 years of disease duration [10]. Our study, describing clinical diagnoses without pathologic confirmation, shows that greater duration of parkinsonism is associated with less likelihood of changes in diagnosis, demonstrating once again that diagnostic accuracy improves with greater duration of parkinsonian symptoms.

A systematic review and meta-analysis by Rizzo et al. showed that the expertise of the clinician affects diagnostic accuracy in PD [6]. These studies used pathologic examination as the gold standard. The diagnostic accuracy was 73.8% by nonexperts, whereas the accuracy was 79.6% by movement disorder specialists, increasing to 83.9% with follow-up. Diagnosis by movement disorder specialists had a sensitivity of 81.3% and a specificity of 83.5%. Diagnosis by nonmovement disorder specialists was more sensitive (89.7%) but less specific (49.2%). Our study was conducted in a tertiary movement disorders center with diagnoses made by movement disorder specialists. Without pathologic confirmation, our results show that movement disorder specialists changed their diagnoses in 6.1% of patients.

The Rizzo study showed that PD diagnoses made by movement disorder specialists are more accurate than diagnoses made by non-movement disorder specialists. While clinicopathological studies show that the most common misdiagnoses are other neurodegenerative forms of parkinsonism, including PSP and MSA [10], our clinical study shows that the most common cause of diagnostic switches to or from PD was drug-induced parkinsonism, followed by MSA, PSP, and DLB. Rare causes of diagnostic switches were vascular parkinsonism and CBS. The difficulty of distinguishing PD from DIP is an important finding in this study. This underscores the importance of a thorough medication history and also highlights the difficulty distinguishing DIP from an exacerbation of an emerging parkinsonian disorder. Dopamine transporter imaging is a useful diagnostic test in this clinical setting since it distinguishes between PD and DIP. Since DIP often takes many months to reverse, dopamine transporter imaging may prevent a long delay in PD diagnosis [13].

Why is the frequency of diagnostic switching in this study so low relative to the clinicopathologic studies? The sample population was large (1393 patients) with a mean PD duration of 4.5 years at the first visit and a mean follow-up of 5.4 years. Therefore, the movement disorder neurologists had the advantage of clinical impressions made over the first decade since the onset of parkinsonism in the majority of these patients. Without pathologic confirmation, some of the clinically confirmed diagnoses are likely to be incorrect. However, the large majority of PD patients never have pathologic confirmation. Therefore, the stability of clinical diagnoses and the common causes of misdiagnosis are important information for clinical neurologists.

This study has some limitations. While previous studies confirmed PD diagnoses with pathologic correlation, in our study diagnoses were made based on established clinical criteria. Our clinical database does not include data on diagnostic investigations, so we were unable to analyze the relationship between tests including brain MRI and diagnostic accuracy. These limitations are balanced by the strength of data from a naturalistic clinical setting. The data were collected prospectively over a 15-year period (2002–2017); however, patients who received only a single consultation were excluded from the study since follow-up data were not available. Some study patients were lost to follow-up after a few visits, before establishing a diagnosis. This study focuses on changes in the clinical diagnosis of PD among movement disorder specialists. Patients with continued diagnostic uncertainty are not the focus of this study and were not included. This study was conducted in a tertiary movement disorder center, a potential source of ascertainment bias, as patients were often seen by other physicians first.

In summary, this study shows that movement disorder specialists in an academic tertiary care center changed the clinical diagnosis of PD in 6.1% of patients over 15 years of clinical practice. The most common causes of diagnostic switches, to or from PD, were drug-induced parkinsonism, MSA, PSP, and DLB. Our data show that experienced movement disorder specialists do not commonly revise their clinical diagnosis and that DIP is the most common cause of changing the diagnosis over time. Patients with shorter disease duration but greater disease severity and greater comorbidity were more likely to be misdiagnosed at the initial office visit. Helpful tips for neurologists with less experience in movement disorders include having a higher index of suspicion when seeing patients with the combination of relatively short disease duration with significant symptoms and disability, as well as patients with multiple comorbid conditions that may obscure the neurologic presentation. The results of this study show the importance of confirming the diagnosis of PD at subsequent clinical encounters [1].

## Figures and Tables

**Table 1:** Characteristics of patients by stability of diagnosis.

Characteristics of patients by stability of diagnosis	Total (*n* = 1393)	No Dx change (*n* = 1308)	Dx change (*n* = 85)
Age at the first visit (years), mean (sd)	70.3 (10.3)	70.3 (10.3)	70.4 (10.5)
Gender, *n* (column percent)			
Male	884 (63.9)	824 (63.4)	60 (70.6)
Female	500 (36.1)	475 (36.6)	25 (29.4)
Race, *n* (column percent)			
White	1125 (90.7)	1055 (90.5)	70 (93.9)
Nonwhite	116 (9.3)	111 (9.5)	5 (6.7)
Marital status, *n* (column percent)			
Married	986 (71.2)	924 (71.0)	62 (73.8)
Not married	399 (28.8)	377 (29.0)	22 (26.2)
Education, *n* (column percent)			
College or more	918 (73.6)	866 (73.7)	52 (72.2)
Less than college	329 (26.4)	309 (26.3)	20 (27.8)
Income, *n* (column percent)			
<50 K/year	244 (25.3)	226 (25.0)	18 (29.0)
>50 K/year	722 (74.7)	678 (75.0)	44 (71.0)
Employment status, *n* (column percent)			
Not employed	826 (59.6)	771 (59.2)	55 (65.5)
Employed	561 (40.4)	532 (40.8)	29 (34.5)
PD duration at the first visit (years), m (sd)	4.5 (5.2)	4.6 (5.3)	3.1 (5.0)
PD duration at follow-up (years), m (sd)^*∗*^	5.4 (3.8)	5.4 (3.8)	4.9 (3.7)
First CIRS-G total score, m (sd)^*∗∗∗*^	4.9 (3.7)	4.8 (3.6)	6.4 (4.0)
First UPDRS motor score, m (sd)^*∗∗*^	26.2 (12.6)	26.0 (12.6)	29.8 (12.4)
First UPDRS total score, m (sd)^*∗*^	38.7 (18.4)	38.4 (18.4)	43.6 (18.3)
Change of the treating neurologist, n (column percent)			
No	807 (84.6)	757 (84.5)	50 (86.2)
Yes	147 (15.4)	139 (15.5)	8 (13.8)
Levodopa at the first visit, *n* (column percent)			
No	813 (58.4)	760 (58.1)	53 (62.4)
Yes	580 (41.6)	548 (41.9)	32 (37.6)
Levodopa at the last visit, *n* (column percent)			
No	626 (44.9)	588 (45.0)	38 (44.7)
Yes	767 (55.1)	720 (55.0)	47 (55.3)
Dopaminergic Rx at the first visit, *n* (column percent)			
No	658 (47.2)	613 (46.9)	45 (52.9)
Yes	735 (52.8)	695 (53.1)	40 (47.1)
Dopaminergic Rx at the last visit, *n* (column percent)			
No	579 (41.6)	546 (41.7)	33 (38.8)
Yes	814 (58.4)	762 (58.3)	52 (61.2)
Dyskinesia at the first visit^*∗∗∗*^			
No	1064 (80.5)	986 (79.5)	78 (95.2)
Yes	258 (19.5)	254 (20.5)	4 (4.9)
Fluctuator at the first visit^*∗∗*^			
No	953 (71.8)	882 (70.8)	71 (81.6)
Yes	374 (28.2)	363 (29.2)	11 (13.4)
MMSE at the first visit, mean (sd)	28.5 (2.1)	28.5 (2.0)	28.1 (2.7)

^*∗*^ *p* < 0.05, ^*∗∗*^*p* < 0.01, and ^*∗∗∗*^*p* < 0.001.

**Table 2:** Patient characteristics by direction of change in diagnosis.

Patient characteristics by direction of change in diagnosis	PD to parkinsonism (*n* = 31)	Parkinsonism to PD (*n* = 27)	Multiple switches (*n* = 27)
Age at the first visit (years), m (sd)	68.7 (12.5)	70.3 (9.4)	72.6 (9.0)
Gender, *n* (column percent)			
Male	22 (71.0)	18 (66.7)	20 (74.1)
Female	9 (29)	9 (33.3)	7 (25.9)
Race, *n* (column percent)			
White	25 (92.6)	22 (91.7)	23 (95.8)
Nonwhite	2 (7.4)	2 (8.3)	1 (4.2)
Marital status, *n* (column percent)			
Married	21 (70.0)	20 (74.1)	21 (77.8)
Not married	9 (30.0)	7 (25.9)	6 (22.2)
Education, *n* (column percent)			
College or more	20 (74.1)	15 (71.4)	17 (70.8)
Less than college	7 (25.9)	6 (28.6)	7 (29.2)
Income, *n* (column percent)			
<50 k/year	10 (43.5)	3 (17.6)	5 (22.7)
50 k+/year	13 (56.5)	14 (82.4)	17 (77.3)
Employment status, *n* (column percent)^*∗*^			
Not employed	15 (50.0)	18 (66.7)	22 (81.5)
Employed	15 (50.0)	9 (33.3)	5 (18.5)
Duration of parkinsonism at the first visit (years), m (sd)	1.7 (2.9)	3.9 (5.7)	3.9 (6.0)
Duration of parkinsonism at follow-up (years), m (sd)	3.7 (2.7)	5.6 (4.1)	5.6 (3.8)
First CIRS-G total score, m (sd)	5.3 (3.0)	6.3 (4.8)	7.8 (4.0)
First UPDRS motor score, m (sd)	27.6 (10.0)	30.6 (14.7)	31.7 (12.5)
First UPDRS total score, m (sd)	38.7 (14.9)	44.5 (21.5)	48.1 (17.5)
Change of the clinician, *n* (column percent)			
No	18 (85.7)	15 (93.8)	17 (81.0)
Yes	3 (14.3)	1 (6.3)	4 (19.0)
Levodopa at the first visit, *n* (column percent)			
No	19 (61.3)	18 (66.7)	16 (59.3)
Yes	12 (38.7)	9 (33.3)	11 (40.7)
Levodopa at the last visit, *n* (column percent)			
No	12 (38.7)	15 (55.6)	11 (40.7)
Yes	19 (61.3)	12 (44.4)	16 (59.3)
Dopaminergic Rx at first visit, *n* (column percent)			
No	16 (51.6)	14 (51.9)	15 (55.6)
Yes	15 (48.4)	13 (48.1)	12 (44.4)
Dopaminergic Rx at the last visit, *n* (column percent)			
No	10 (32.3)	13 (48.1)	10 (37.0)
Yes	21 (67.7)	14 (51.9)	17 (63.0)

^*∗*^ *p* < 0.05.

## Data Availability

Further data are available on request to the corresponding author.
